# Philosophy in Homœopathy

**Published:** 1890-08

**Authors:** 


PHILOSOPHY IN HOMŒOPATHY. Addressed to the Medical Profession and the General Reader. By Chas. S. Mack, M. D., Prof. Materia Medica and Therapeutics, in the Homœopathic Medical College, of the University of Michigan, at Ann Arbor. Chicago : Gross & Delbridge. 1890.

Partisan judgment, like partisan legislation, is productive of much error and bad feeling. The author of this work indicates the sentiment of the fanatic, who "*had rather die under the treatment of* SIMILIA, *than get well under the chance* of CONTRARIA." Certainly it is not philosophy to indulge in these prejudices, while excluding the broad and liberal principles of reason, truth and honesty. All schisms are wrong in detail, but the exercise of a simple charity, and the practice of common honesty, would create a new order of things, and the "Radical Physician" would represent the sum total of the "*practical catching*" diversities of name of this day and generation. Relegate the Homœopath, Allopath, Hydropath, etc, with the "*advertising* quack" to the realm of humbuggery, and let all the honest and conscientious members of the body medical co-operate on the broad basis of *rational medicine !*

We have very little patience with these class dissertations, long winded and empty, and long for the time when unity, good will, science and *common sense* shall characterise the literary and practical efforts of our professional brethren.

ROBT. W. W.

